# Co-incidence of RCC-susceptibility polymorphisms with HIF cis-acting sequences supports a pathway tuning model of cancer

**DOI:** 10.1038/s41598-019-55098-7

**Published:** 2019-12-10

**Authors:** Virginia Schmid, Veronique N. Lafleur, Olivia Lombardi, Ran Li, Rafik Salama, Leandro Colli, Hani Choudhry, Stephen Chanock, Peter J. Ratcliffe, David R. Mole

**Affiliations:** 1 Ludwig Institute for Cancer Research, University of Oxford, Old Road Campus, Headington, Oxford, OX3 7FZ United Kingdom; 2 NDM Research Building, University of Oxford, Old Road Campus, Headington, Oxford, OX3 7FZ United Kingdom; 3 Division of Cancer Epidemiology and Genetics, room 7E412, MSC 9776, National Cancer Institute, 9609 Medical Center Drive, Bethesda, MD 20892-9776 USA; 4 Department of Biochemistry, Faculty of Science, Center of Innovation in Personalized Medicine, King Fahd Center for Medical Research, King Abdulaziz University, Jeddah, Saudi Arabia; 5 The Francis Crick Institute, 1 Midland Road, London, NW1 1AT United Kingdom; 6 NIHR Oxford Biomedical Research Centre, Oxford University Hospitals NHS Foundation Trust, John Radcliffe Hospital, Oxford, OX3 9DU United Kingdom

**Keywords:** Urological cancer, Cancer genetics

## Abstract

Emerging evidence suggests that dysregulation of oncogenic pathways requires precise tuning in order for cancer to develop. To test this, we examined the overlap between cis-acting elements of the hypoxia-inducible factor (HIF) pathway and cancer-susceptibility polymorphisms as defined in genome-wide association studies (GWAS). In renal cancer, where HIF is constitutively and un-physiologically activated by mutation of the von Hippel-Lindau tumour suppressor, we observed marked excess overlap, which extended to potential susceptibility polymorphisms that are below the conventional threshold applied in GWAS. In contrast, in other cancers where HIF is upregulated by different mechanisms, including micro-environmental hypoxia, we observed no excess in overlap. Our findings support a ‘pathway tuning’ model of cancer, whereby precise modulation of multiple outputs of specific, activated pathways is important in oncogenesis. This implies that selective pressures to modulate such pathways operate during cancer development and should focus attempts to identify their nature and consequences.

## Introduction

The combined study of the genetics and the molecular cell biology of cancer has transformed our understanding of the disease. Much of this work concerns the role of major tumour suppressors and oncogenes and has led to a now classical model in which mutational dysregulation of these molecules generates a discrete effect on a specific pathway that directly promotes the development of cancer^1–3^. However, despite these insights, many aspects of the disease remain perplexing. For example, although these pathways frequently operate widely, genetic studies have repeatedly revealed highly constrained patterns of mutation that are difficult to understand under such a model^4–6^. Indeed, the specificity of oncogenic mutations, both within individual proteins^7^ and within the components of a functional complex^8^, together with the context and tissue specificity of mutations that affect proteins with general cellular functions^9^, are all difficult to explain solely by reference to the inactivation or activation of a single mechanistic pathway acting in isolation.

At the same time, continued investigation of many oncogenic pathways has revealed much greater complexity than was foreseen when they were first defined. In particular, pan-genomic analyses, using high-throughput sequencing methods, have revealed extra-ordinary complexity in gene regulatory and transcriptional outputs^10,11^. To date, the complexity of these networks has received relatively little attention in theories of cancer evolution. However, the existence of extensive interconnected pathways, predicts that the mutational dysregulation of a pathway will lead to multiple effects that are unlikely to be neutral with respect to cancer development. A model in which multiple effects that may differ in sign and size with respect to cancer development is easier to reconcile with the functional precision that is implied by highly constrained patterns of mutation. Under such a model the context-specific summation of (potentially) large numbers of positive and negative influences would determine a ‘net’ oncogenic balance. If true, this is of interest since it would also imply that relatively modest selective pressures on multiple components of interconnected pathways should be important during cancer evolution.

We have sought to investigate this from the perspective of the transcriptional response to hypoxia, which is mediated by hypoxia-inducible factor (HIF). HIF is commonly activated in cancer, either by the hypoxic tumour microenvironment or through connection with oncogene or tumour suppressor pathways^12,13^. Of these, the most striking is the constitutive activation of HIF that follows bi-allelic inactivation of the von Hippel-Lindau tumour suppressor (pVHL) in renal clear cell carcinoma (RCC), the most prevalent form of kidney cancer^14,15^. pVHL is the recognition component of a ubiquitin E3 ligase that functions physiologically to degrade HIF in the presence of oxygen^16,17^. In VHL-defective RCC, this process is blocked, leading to un-physiological activation of HIF even in well oxygenated cells. Pan-genomic investigations of the HIF pathway reveal hundreds to thousands of cell-type and/or context-specific direct HIF transcriptional targets, whose effects on gene expression are extended by the indirect actions of secondary transcriptional cascades^18–21^. *A priori*, it might be expected that when such an extensive pathway is activated un-physiologically, then the numerous alterations in gene expression would have heterogeneous actions on oncogenesis, with selective pressure operating to modulate or tune these outputs during the evolution of the cancer.

Several pieces of evidence support this pathway-tuning model. Firstly, HIF exists as several different isoforms, composed of separate HIF-α subunits dimerised with a common HIF-1β subunit. In RCC, experimental evidence largely supports the tumour promoting activity of HIF-2, but not HIF-1^22,23^. This suggests that the increased expression of HIF-2α, which is observed in neoplastic as opposed to normal renal tubular cells^24^, represents modulation of the activated HIF pathway to a more oncogenic form. Secondly, heterogeneous associations of specific HIF-1 and HIF-2 target genes with RCC prognosis suggests that within the transcriptional repertoire of each isoform, there may be multiple bidirectional influences on RCC oncogenesis^25^. However, small heterogeneous effects that are likely to be contextually specific are difficult to prove statistically by reference to somatic genetics of RCC, and to assay functionally.

However, we have observed that certain germline RCC-susceptibility polymorphisms, defined by individual genome-wide association studies (GWAS), influence HIF-binding sites and alter expression of their target genes in RCC cell lines^26–28^. GWAS are potentially informative for the proposed pathway-tuning model since they examine for association between a biologically relevant output, (the development of clinically overt RCC), and a standardised set of SNPs that can be specified in large numbers of individuals^29^. Although GWAS approaches require large numbers of patients to achieve sufficient power and lack the ability to define the exact extra-genic cis-acting sequences on which such polymorphisms might operate, when combined with pan-genomic transcriptional analyses, they potentially provide a powerful tool to study the effects of pathway tuning on the development of renal cancer. Therefore, we have sought to examine, as far as is possible, the extent of overlap between cancer-susceptibility polymorphisms and the HIF cis-acting apparatus, taking advantage of several large recently published cancer GWAS^30–33^.

The work demonstrates striking excess overlap between RCC-associated polymorphism and the cis-acting elements in the HIF transcriptional system that is statistically robust under different definitions of these elements. This association extends to RCC-associated polymorphisms that are below conventional thresholds for statistical significance used in GWAS. The excess overlap with HIF-binding elements was specific for RCC and was not seen in other cancers, including breast and prostate cancer cell lines where, instead, excess overlap with oestrogen and androgen receptor binding sites was observed. These results support a model in which the precise tuning of the multiple outputs of an activated pathway is required to promote oncogenesis. This in turn creates selective pressures that are reflected in human cancer susceptibility polymorphisms.

## Results

### Overlap between RCC susceptibility polymorphisms and cis-acting elements of the HIF pathway

We first sought to determine the presence or otherwise of significantly excess overlap between RCC-associated human polymorphisms and HIF-binding sites using a recent large meta-analysis of RCC GWAS^30^. To this end, SNP-level summary statistics from that study were used to define loci that reached conventional thresholds (p ≤ 5 × 10^−8^) for genome-wide significance (Table 1). We then identified all SNPs in strong linkage (r^2^ ≥ 0.8) with the index SNP at each locus using the 1,000 Genomes CEU data. These analyses defined discreet series of SNPs at 13 loci, which we refer to as supra-threshold RCC-associated loci. HIF-1α, HIF-2α and HIF-1β binding sites were determined in RCC4 and 786-O RCC cell lines using chromatin immunoprecipitation (ChIP)-seq analysis^25,34^. To provide a stringent definition for the assessment of overlap, only sites with identified ChIP-seq peaks in 2 or more of the 5 datasets (786-O cells do not express functional HIF-1α) were defined as HIF binding sites.

**Table 1:** RCC-susceptibility loci (p-value ≤ 5 × 10^−8^) and their relation to HIF-binding sites and VHL-regulated genes.

Locus	Index SNP	P-values	Proximity to HIF-binding site	Proximal and interacting protein-coding genes	Regulation by VHL/HIF pathway
11q13.3	rs11263654	1.65E-23	overlap	CCND1	↑
				MYEOV	ne
				ORAOV1	↑
2p21	rs2121267	6.95E-19		EPAS1	↓
12p12.1	rs11534749	6.19E-17	overlap	BHLHE41	—
				ITPR2	↑
				SSPN	ne
14q24.2	rs28840762	1.08E-15		DPF3	ne
12q24.31	rs10846748	1.70E-12	<25 kb	SCARB1	↑
8q24.21	rs6470588	2.82E-12	overlap	MYC	↑
11q22.3	rs117706999	6.60E-10		DDX10	↑
				EXPH5	ne
				ATM	↑
				NPAT	—
				C11orf65	ne
				KDELC2	↓
2q22.3	rs11888238	8.15E-10		ZEB2	↑
1p32.3	rs6676515	3.04E-09		FAF1	—
				DMRTA2	ne
4q23	rs7697932	4.70E-09	<25 kb		
3p22.1	rs9821249	1.12E-08		EIF1B	↓
				ENTPD3	ne
				RPL14	↓
				ZNF619	↑
				ZNF620	ne
				ZNF621	↑
15q22.31	rs12905354	2.28E-08	<25 kb	DIS3L	—
				MAP2K1	↑
				MEGF11	ne
				SMAD6	—
				SNAPC5	↓
				TIPIN	↑
				ZWILCH	↑
				RPL4	—
3q26.31	rs234043	4.10E-08	overlap	ECT2	↑
				TNFSF10	↑

Table shows each RCC-susceptibility locus, together with the most-significant (index) SNP at that locus and the significance level associated with that SNP. The proximity (direct overlap or within 25 kb) to a HIF-binding site is indicated. Protein-coding genes within 25 kb of each RCC-susceptibility locus, together with those that are more distant, but which show promoter (TSS +/− 2 kb) interaction in Capture-C analyses, are listed. Differential expression of each of these genes in VHL-deficient versus VHL-transfected 786-O cells is shown (↑ = significantly upregulated in VHL-deficient cells, ↓ = significantly downregulated in VHL-deficient cells, — = not regulated, ne = not expressed, fdr ≤ 0.05).

**Figure 1:**
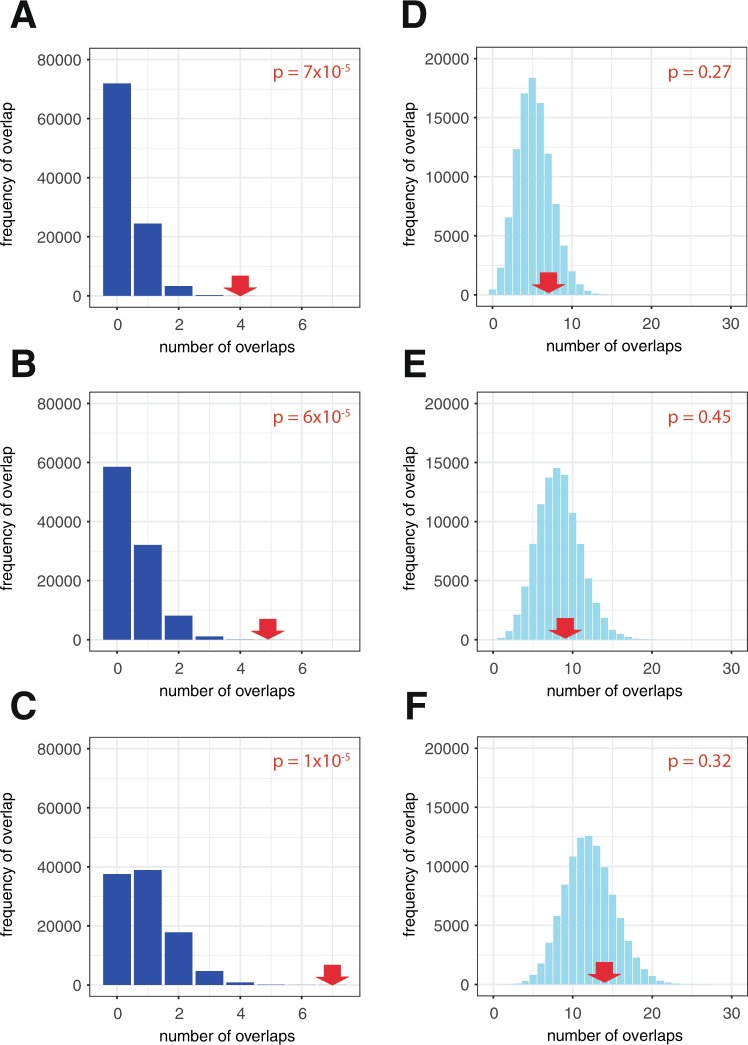
RCC-associated polymorphic loci overlap HIF-binding sites in RCC cells. (**A**) RCC-associated polymorphisms overlap HIF-binding sites in RCC cell lines. 4 out of 13 GWAS loci directly overlapped HIF-binding sites in RCC cells (red arrow). The blue bars denote the frequency distribution of the expected overlap in the bootstrapping approach when the GWAS loci were randomly shuffled around the genome 100,000 times. Significant enrichment (p = 7 × 10^−5^) of active enhancers at RCC-associated GWAS loci was observed. (**B**) HIF-binding enhancers were targeted in preference to those that do not bind HIF. The converse bootstrapping approach in which the HIF-binding sites were randomly shuffled, but constrained to regions defined as active enhancers, again showed that this overlap was significantly greater than expected (p = 6 × 10^−5^). Note that one of the RCC-susceptibility loci directly overlapped two HIF-binding sites. (**C**) RCC-associated polymorphisms are enriched in proximity to HIF-binding sites. 7 GWAS loci either overlapped or lay within 25 kb of a HIF-binding site. A similar bootstrapping approach, in which the number of shuffled loci lying within 25 kb of a HIF-binding site was used to determine the significance of the observed result (p = 1 ×10^−5^). (**D**–**F**) For comparison, the same analyses were performed using GWAS loci associated with educational achievement, which showed no significant enhancement for overlap with HIF-binding sites.

At 4 out of the 13 loci (11q13.3, 8q24.21, 12p12.1 and 3q26.31), an RCC-associated SNP directly overlapped a HIF-binding site. To determine whether this overlap is significantly greater than expected by chance, we employed a bootstrapping approach. The RCC-associated GWAS loci were randomly shuffled around the genome 100,000 times and the number overlapping a HIF-binding site was used to construct a frequency distribution for the expected number of overlaps (Fig. 1A). This defined a probability of randomly observing 4 loci overlapping a HIF-binding site of 7 × 10^−5^, indicating that RCC-susceptibility loci are indeed significantly enriched for HIF-binding sites. However, since GWAS loci, generally, are known to be enriched at enhancer sites^35^ and since HIF binds to enhancers, it is possible for increased overlap to arise from general enrichment for enhancers, rather than representing a specific overlap between RCC-associated loci and regulatory elements affecting the HIF pathway. To resolve this, we randomly shuffled HIF-binding sites within regions defined as enhancers^36^, on the basis of ChIP-seq signals for histone H3K4me1, H3K4me3 and H3K27ac modification in 786-O RCC cells, and again tested for excess overlap with RCC-susceptibility loci. A similar bootstrapping approach using 100,000 iterations revealed a probability of observing overlap with 5 HIF-binding sites by chance of 6 × 10^−5^; note one locus overlaps two HIF-binding sites (Fig. 1B).

The above findings confirm that RCC-associated polymorphisms are significantly enriched at cis-acting elements mediating the HIF transcriptional output, as defined by direct overlap with ChIP-seq signals for HIF binding. Although the entirety of cis-acting sequences affecting the HIF transcriptional cascade cannot be defined with precision, at any given locus it clearly extends beyond the HIF-binding site itself. We therefore sought to test whether the excess overlap with RCC-susceptibility polymorphisms might extend more widely at HIF-binding loci, and repeated the overlap analysis using an extended sequence of +/− 25 kb from the HIF binding peak identified by ChIP-seq. A further three RCC-susceptibility loci (12q24.31, 4q23 and 15q22.31) lay within these sequences; the same bootstrapping approach demonstrated a similar level of statistical significance (p = 1 × 10^−5^) for this overlap with RCC-susceptibility loci as was obtained using direct overlap with the HIF ChIP-seq signal itself (Fig. 1C).

For comparison, we repeated each of these analyses using susceptibility polymorphisms for an unrelated control phenotype (educational attainment) from the National Human Genome Research Institute – European Bioinformatics Institute (NHGRI-EBI) GWAS catalogue^37^. No excess overlap with HIF-biding sites, or with the extended loci was observed (Fig. 1D–F). Taken together, these analyses confirm that RCC-associated polymorphisms overlap HIF binding sites more frequently than expected by chance, demonstrate that this is likely to reflect, at least in part, a specific association with the HIF transcriptional apparatus and suggest that significant overlap may extend to sequences at HIF target gene loci that lie beyond those identified as directly binding HIF in ChIP-seq analyses. In addition, although not considered as a HIF target gene in the above analyses, another of the thirteen supra-threshold RCC-associated loci, 2p21, includes *EPAS1*, the gene encoding HIF-2α.

**Figure 2:**
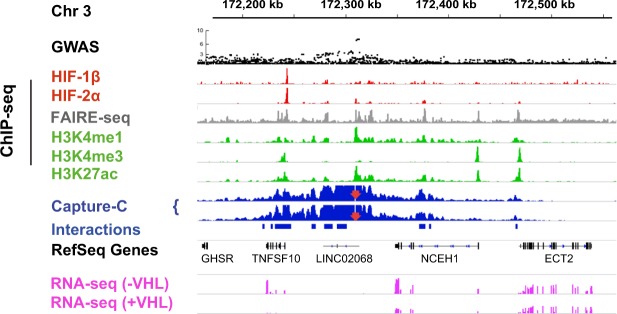
RCC-susceptibility polymorphisms at the 3q26.31 locus loop to a HIF-binding site at the *TNFSF10* promoter. Integrative Genomics Viewer (IGV) tracks showing GWAS SNP-level p-values from the RCC GWAS meta-analysis at the 3q26.31 locus together with HIF binding (red tracks), histone modifications (green tracks), chromatin structure (FAIRE-seq – grey track and Capture-C – blue tracks) and RNA-seq analysis (pink tracks) in 786-O RCC cells transfected with wild-type VHL (+VHL) or untransfected (-VHL). The locus overlaps a weak HIF-2α ChIP-seq peak. However, this region shows long-distance chromatin looping to a much stronger HIF peak close to the *TNFSF10* promoter. The red arrow denotes the “viewpoint” used in the Capture-C analysis. Chromosomal coordinates and gene annotation are from the RefSeq hg19 (GRCh37) build.

To gain a more precise view of regions of the genome that physically interact with each RCC-susceptibility locus, we next analysed chromatin looping from each locus using Capture-C. This method has the ability to detect physical interactions with additional enhancers that lie outside the locus itself, as well as with the promoters of putative target genes^38^. ChIP-seq analysis of histone H3K4me1, H3K4me3 and H3K27ac modifications together with formaldehyde-assisted isolation of regulatory elements (FAIRE)-seq analysis of DNA accessibility in 786-O RCC cells was used to identify functional elements within each supra-threshold RCC-susceptibility locus. One RCC-associated locus did not contain any such sites in 786-O cells and was not studied further. Capture-C analysis was then performed using ‘view-point’ oligonucleotides that targeted each of the functional elements at all 12 loci (Supplemental Figs. 1 and 2). This analysis revealed that the 2p21 (*EPAS1*) locus also made long-range chromatin interaction with a HIF-binding site (Supplemental Fig. 1B). In addition, the 3q26.31 locus was observed to interact with another, much stronger HIF-binding site lying close to the promoter of the *TNFSF10* gene (Fig. 2). Conversely, each of the three other loci that overlapped directly with HIF-binding sites (11q13.3, 8q24.21, 12p12.1) also showed long-range physical interactions with other weaker HIF ChIP-seq peaks, which may result from co-immunoprecipitation of looped sites with the actual HIF-binding site. This indicates that in addition to affecting HIF-binding sites directly, RCC-associated polymorphisms can affect other enhancers that physically interact with HIF-binding sites to regulate a common transcriptional target. In total, 8 out of the 13 loci associated with RCC, either directly overlap, are close to a HIF-binding site, or physically interact with a distant HIF-binding site in RCC cell lines and therefore share the potential to affect the expression of a HIF target gene.

**Figure 3:**
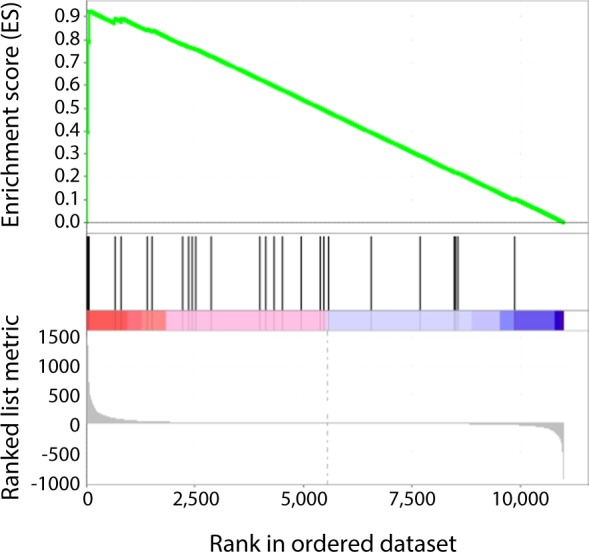
RCC-susceptibility loci are enriched at regions that are close to or physically associated with HIF regulated genes. RNA-seq analysis (n = 3) of 786-O (VHL-defective) cells and 786-O cells stably transfected with wild-type *VHL* was used to rank all measurable genes according to their regulation (combined fold-change and p-value) by VHL (x-axis). Genes with promoters lying within 25 kb of each RCC-susceptibility locus, or more distant genes whose promoters were shown to loop to these loci in Capture-C analyses, were examined and are illustrated as vertical bars. Weighted gene set enrichment analysis (green line) was used to test for association and showed significant enrichment of this gene set amongst those that are upregulated, but not down regulated in VHL-defective cells (p = 0.017).

To pursue this, we examined for an association between these loci and the genes that are regulated by the VHL/HIF pathway. The Capture-C methodology cannot reliably resolve interactions over short physical distances. In this analysis, we therefore included all genes that lay within 25 kb of the Capture-C ‘viewpoint’ oligonucleotide at each RCC-susceptibility locus, as well as those whose promoters were more distant, but exhibited physical interaction with the RCC-susceptibility locus in the Capture-C analyses. This revealed 36 genes whose promoters might potentially be direct targets of enhancers at each of the RCC-associated loci (Table 1). To identify genes regulated by the HIF pathway, we then performed poly-adenylated RNA-seq analysis of 786-O cells stably transfected either with wild-type VHL or with control vector. Genes were ranked according to their differential expression in VHL-deficient versus VHL-competent cells. Gene Set Enrichment Analysis (GSEA) of the 36-gene set showed enrichment for genes that are upregulated in VHL defective cells (Fig. 3). In summary, these analyses show that RCC-associated loci are strongly enriched for both cis-acting elements of the HIF apparatus, and for transcriptionally enhanced targets of the VHL/HIF pathway.

**Figure 4:**
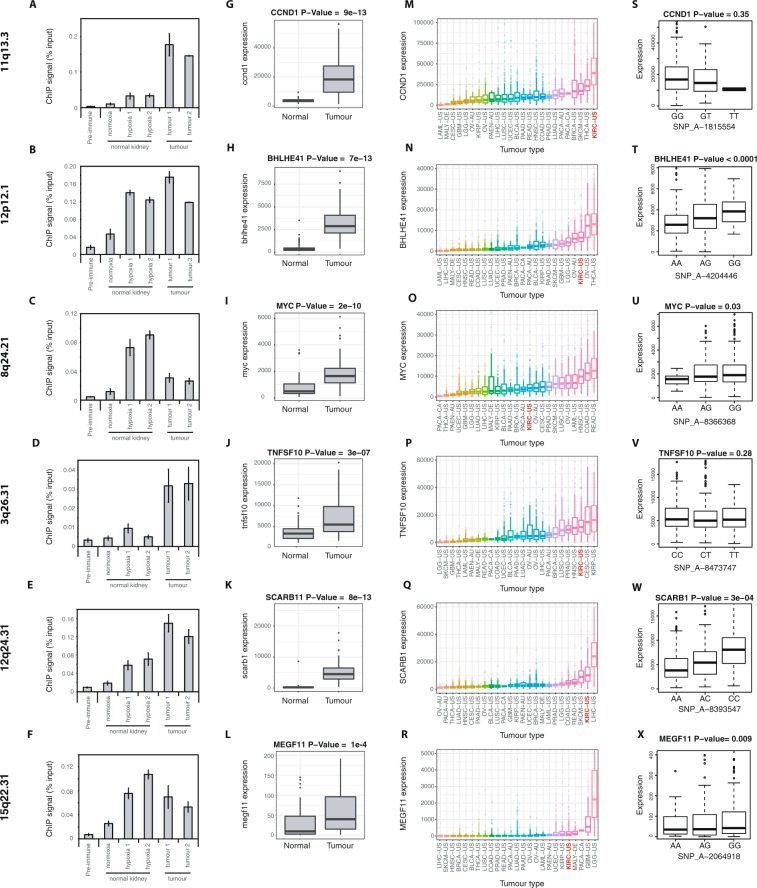
HIF binding and gene expression at RCC-susceptibility loci in RCC tissues. (**A**–**F**) Freshly explanted cells from a ccRCC tumour and from the surrounding normal kidney were grown in primary culture. HIF-1β ChIP-qPCR analysis of HIF-binding sites that overlapped or interacted with RCC-susceptibility loci confirmed the binding observed in ChIP-seq analysis of RCC cell-lines (N.B. data shown for the 3q26.31 locus is for the strong HIF-binding site close to the TNFSF10 promoter). (**G**–**L**) Box-and-whisker plots showing the expression level of genes, interacting with the HIF-binding site at each locus, in ccRCC tissue and surrounding normal kidney using paired RNA-seq data from the TCGA KIRC cohort. Statistical significance was determined using a paired Wilcoxon rank sum test. (**M**–**R**) Box-and-whisker plots comparing the expression level of each of these genes in the TCGA KIRC cohort to that observed in RNA-seq analyses of other tumour types in the TCGA database. (**S**–**X**) Patients in the TCGA KIRC cohort were sub-divided according to their germline genotype at each RCC-susceptibility locus. Box-and-whisker plots show the correlation between the genotype and the expression level of each of the genes in ccRCC tissue. The significance of the association between the SNP genotype and gene expression was determined by fitting RNA-Seq expression across patients to a negative binomial Generalized Linear Model (GLM) against the genotype status. The likelihood ratio of this model versus a model that ignores genotype status was then computed and a Chi-Square test used to call significance of the genotype coefficients in stratifying the patients.

To test the relevance of these findings in renal tumours themselves, we next performed HIF-1β ChIP-qPCR analysis of each of the HIF-binding sites that overlapped or interacted with a RCC-susceptibility locus, using primary cultures of tumour and normal cells explanted from a patient with kidney cancer (Fig. 4A–F). This confirmed binding of HIF in RCC tumours as well as in the immortalised RCC cell-lines used in the ChIP-seq analyses. For each locus, we then examined the expression levels of each gene that interacted with the HIF-binding site in clear cell kidney tumours (in which HIF is constitutively active) and in paired surrounding kidney (in which HIF is not activated), using RNA-seq analyses from The Cancer Genome Atlas (TCGA) ccRCC (KIRC) cohort. At all 6 loci at which an interacting gene was identified, one or more of these genes showed strong induction in renal tumours compared to normal kidney (Fig. 4G–L). Further analysis of TCGA RNA-seq datasets from multiple tumours types revealed that, each of these genes was highly expressed in ccRCC compared to other cancers (Fig. 4M–R). In addition, when patients in the TCGA KIRC cohort were sub-divided according to their germline genotype, expression of these genes, in renal cancer tissue, frequently correlated with the genotype at each RCC-susceptibility locus (Fig. 4S–X). Finally, polymorphisms at the 11q13.3 locus have been shown to alter binding of HIF to a long-range enhancer of the CCND1 gene^26^, polymorphisms at the 8q24.21 locus alter binding of HIF to an enhancer of MYC^27^, and polymorphisms at the 12p12.1 locus also alter HIF binding and BHLHE41 expression^28^.

Whilst many of these genes have well characterized roles in cancer generally, there are also multiple lines of evidence to show their importance specifically in driving ccRCC. Firstly, in experimental xenograft models of ccRCC, Zhang *et al*.^39^ showed that inhibition of CCND1 in 786-O cells by shRNA slowed tumor growth. Secondly, Tang *et al*.^40^ have shown that the MYC pathway is activated in ccRCC and is essential for the proliferation of human ccRCC cell lines; Shroff *et al*.^41^ have also shown that MYC oncogene overexpression drives renal cell carcinoma in a mouse model; and Bailey *et al*.^42^ also showed that MYC activation cooperates with VHL and INK4A/ARF loss to induce ccRCC in mouse models. Thirdly, Shen *et al*.^43^ have shown that inhibition of BHLHE41 by shRNA inhibits proliferation of A498 and CAKI-1 renal cancer cell lines. Fourthly, in human tumors, high expression of SCARB1 is associated with adverse patient outcomes and in experimental models, inhibition of SCARB1 impairs the proliferation, invasion and migration of ccRCC cells^44^. Finally, although TNFSF10 (TRAIL) has pro-apoptotic actions, in ccRCC, high levels of TNFSF10 correlate with poor patient prognosis and several lines of work have suggested that non-apoptotic functions such as induction of proliferation and cytokine production, as well as influences on immune cells may contribute to the growth of RCC^45^.

### Overlap between cancer-susceptibility loci and HIF-binding sites in different cancer types

**Figure 5:**
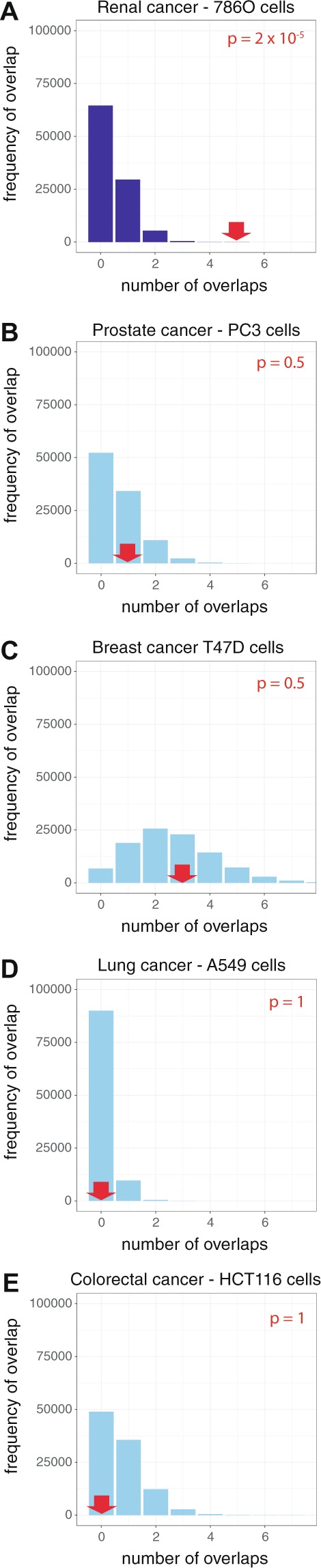
Overlap between HIF-binding sites and GWAS-defined susceptibility loci associated with other cancer types. Cancer-associated polymorphisms from large-scale GWAS analyses were used to define polymorphic loci associated with prostate, breast, lung or colorectal cancer as for RCC. ChIP-seq analysis, in cell lines derived from each type of cancer, using HIF-1β antibodies, was used to define HIF-binding sites. The observed (red arrows) and expected (blue bars) overlap between cancer-associated GWAS loci and HIF-binding sites are shown for: (**A**) RCC GWAS loci and HIF-binding sites in 786-O cells, (**B**) prostate cancer GWAS loci and HIF-binding sites in PC-3 cells, (**C**) breast cancer GWAS loci and HIF-binding sites in T47D cells, (**D**) lung cancer GWAS loci and HIF-binding sites in A549 cells, and (**E**) colorectal cancer GWAS loci and HIF-binding sites in HCT-116 cells. With the exception of RCC (p = 2 ×10^−5^), no significant overlap was observed between cancer-associated GWAS loci and HIF-binding sites in any other cancer type.

Many types of solid tumour manifest increased HIF activity^13,46^. However, activation of the HIF pathway in ccRCC differs in several ways from that observed in other cancer types. Firstly, multiple workers^47–49^ have shown that VHL loss and HIF activation occur at the very earliest stages of ccRCC formation, whereas in other tumour types HIF activation only develops in late stages once the tumour has outgrown its blood supply. Secondly, the activation of HIF in ccRCC is inappropriate to the degree of hypoxia, whereas in other tumour types it is commonly a (patho)physiological response to reduced intra-tumour oxygen levels. This will result in both adaptive and maladaptive responses for well oxygenated ccRCC cells, providing a selection pressure that acts from the earliest stages of ccRCC tumourigenesis to refine the HIF response. In other types of cancer this selection pressure is less marked and of later onset. Therefore, we wished to test whether overlap of cancer-associated polymorphisms with HIF-binding loci extended across other cancer types. To that end we tested for overlap between GWAS-defined susceptibility loci associated with 4 common cancers^31–33^ and HIF-1β binding sites identified in cell lines derived from each of these cancer types. As with the RCC data, SNPs in high linkage (r^2^ ≥ 0.8) with the index SNP at each locus were determined using the 1,000 Genomes CEU data, to define the susceptibility locus. ChIP-seq analysis of HIF-1β binding was performed in duplicate in PC3 prostate cancer cells (970 sites), T47D oestrogen receptor positive (ER+) breast cancer cells (2081 sites), A549 lung cancer cells (1044 sites), and HCT-116 colorectal carcinoma cells (1767 sites). We then examined for overlap between GWAS-defined susceptibility loci and HIF-1β binding sites in each cancer type. To provide direct comparison with the RCC data we repeated the analysis using HIF-1β binding sites defined in the same way in 786-O cells and used the same bootstrap approach to determine the significance of each observed overlap (Fig. 5A–E and Supplemental Table 1). Again, a highly significant (p = 2 × 10^−5^) overlap was observed in kidney cancer. No significant overlap between GWAS-defined cancer-susceptibility loci and HIF-binding sites was observed for any of the other cancer types (p = 0.5 for prostate cancer, p = 0.5 for breast cancer, p = 1 for lung cancer, p = 1 for colorectal cancer).

### Sub-threshold RCC-susceptibility loci associated with RCC are enriched for HIF-binding sites in RCC cells

**Figure 6:**
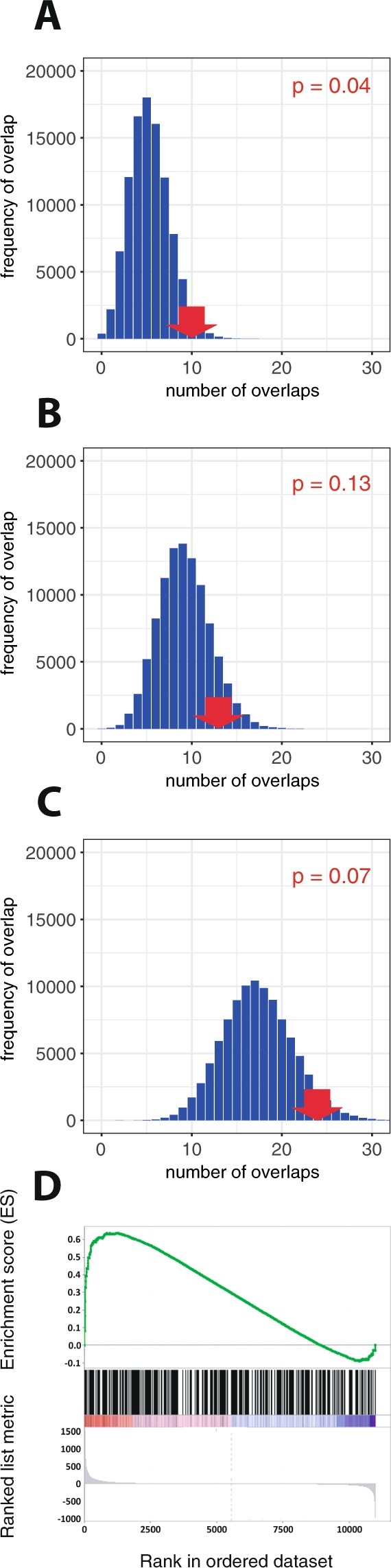
Sub-threshold RCC-associated GWAS loci are enriched at HIF-binding sites and for proximity to HIF-induced genes. Sub-threshold RCC-associated loci were defined as independent GWAS loci at which the index SNP failed to reach genome-wide significance (5 ×10^−8^ ≤ p ≤ 1 ×10^−4^). (**A**) The number overlapping (red arrows) with HIF-binding sites together with the expected overlap (blue bars) was determined as for Fig. 1. (**B**) The converse bootstrapping approach was used in which HIF-binding sites were randomly shuffled but constrained to regions defined as active enhancers. (**C**) The number of sub-threshold loci either overlapping or lying within 25 kb of a HIF-binding site, together with the expected frequency distribution. (**D**) RNA-seq analysis (n =  3) of 786-O cells (stably transfected with wild-type VHL) was used to rank all measurable genes according to their regulation (combined fold-change and p-value) by VHL/HIF (x-axis). The three closest genes to each sub-threshold GWAS locus are illustrated as vertical bars. Weighted gene set enrichment analysis (green line) showed significant enrichment of this gene set amongst upregulated, but not down regulated genes (p = 0.01).

Given the striking overlap of supra-threshold RCC-associated loci with the HIF transcriptional system, we wished to test whether this overlap extended to RCC-associated signals that were below the statistical threshold conventionally used in GWAS^50^. To that end we examined the overlap between RCC-susceptibility loci of intermediate significance (10^−4^ > p > 5 × 10^−8^) and HIF-binding sites. To ensure that these signals represented loci that were independent from above-threshold loci, all SNPs in close physical or genetic proximity (distance <500 Mb or r^2^ ≥ 0.2 in the 1000 Genomes CEU population data) to the index SNP at each above-threshold locus were first excluded. SNP-level summary statistics were then used as above to identify 219 independent loci in this significance window, together with all SNPs in high linkage (r^2^ ≥ 0.8) with the index SNP at each locus, using the 1,000 Genomes CEU data. SNPs at 10 of these sub-threshold loci overlapped with a total of 13 HIF-binding sites in RCC cell lines (Supplemental Table 2). The same bootstrapping approach as for supra-threshold loci indicated that this overlap, which was roughly double the predicted number of loci, was again significantly greater than would be expected by chance, although the significance level (p = 0.04) was less than for the supra-threshold loci (Fig. 6A). However, the probability that a locus of intermediate significance overlapped a HIF-bound enhancer rather than a non-HIF-bound enhancer (Fig. 6B) did not reach statistical significance (p = 0.13). 24 sub-threshold RCC-susceptibility loci either overlapped or lay within 25 kb of a HIF-binding site, which was of borderline significance (p = 0.07) (Fig. 6C). As with the analyses of supra-threshold loci, we compared these results with those of identical analyses conducted on similarly defined susceptibility loci of intermediate significance for educational attainment where no evidence of excess overlap was discernible (Supplemental Fig. 2A–C).

We next examined for an association between sub-threshold RCC-susceptibility loci and genes activated by the VHL/HIF pathway. Genes were again ranked according to their differential expression in VHL- deficient versus VHL-competent cells using the same poly-adenylated RNA-seq analysis of 786-O cells stably transfected either with wild-type VHL or with control vector. We identified the three closest genes to each of the 219 sub-threshold loci. GSEA showed that this gene set was significantly enriched amongst genes up regulated in the absence of VHL (Fig. 6D), (ES = 0.64, NES = 1.34, p = 0.01). Taken together these analyses suggest that RCC-susceptibility polymorphisms extend beyond those currently defined as significant in GWAS, and that at least part of this susceptibility is mediated by polymorphisms that impinge on the VHL/HIF pathway.

### Overlap between cancer-associated susceptibility loci and the binding of other transcription factors

**Figure 7:**
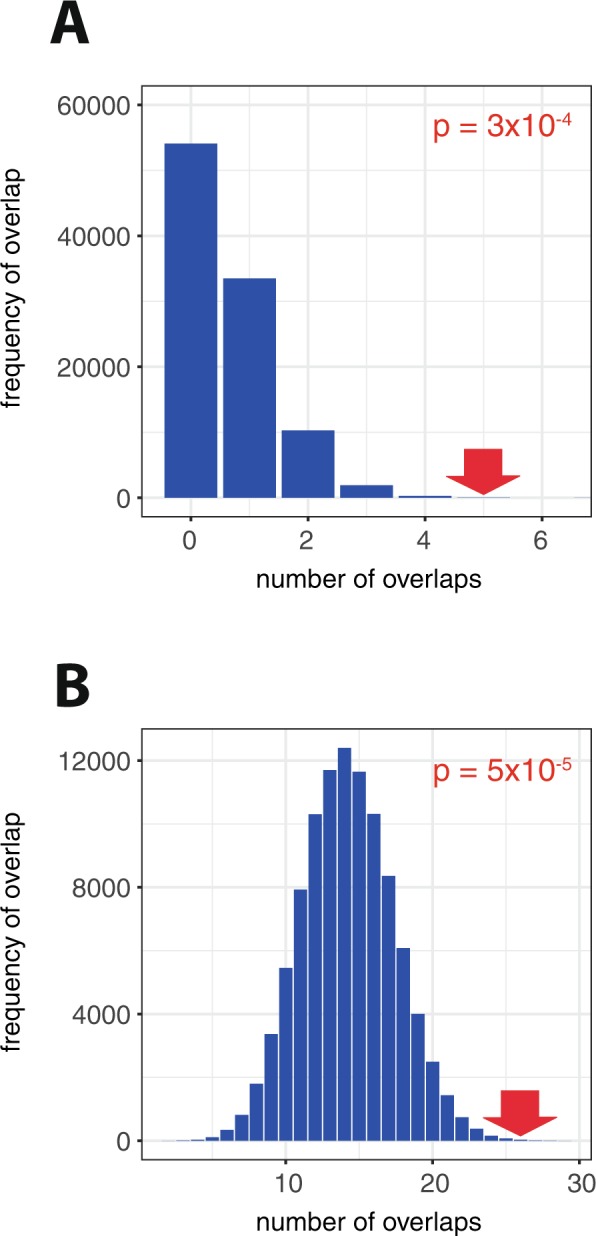
Overlap of cancer-associated GWAS signals and other activated transcriptional pathways. Cancer-associated polymorphisms from large-scale GWAS analyses were used to define polymorphic loci associated with breast or prostate cancer as above. Transcription factor binding sites were defined using ChIP-seq analysis of (**A**) ESR1 binding in T47D cells stimulated with the oestrogen analogue bisphenol A (GSM1010823) and (**B**) AR binding in PC3 cells stimulated with the androgen analogue R1881 (GSE54110). The observed (red arrows) and expected (blue bars) overlap between cancer-associated GWAS loci and the transcription factor binding sites is illustrated as before.

Finally, we sought to test whether cancer-susceptibility polymorphisms might overlap with transcriptional networks that are important in other cancers. To test this, we took advantage of ChIP-seq data from Gene Expression Omnibus (GEO) for oestrogen receptor alpha (ERα or ESR1) binding in T47D breast cancer cells stimulated with the oestrogen receptor agonist bisphenol A (BPA) – 1,878 sites^51^ and for androgen receptor (AR) binding in PC3 prostate cancer cells stimulated with the androgen receptor agonist R1881 – 34,777 sites^52^. Five out of 124 breast cancer-susceptibility loci overlapped with an oestrogen receptor binding site in breast cancer cells (Supplemental Table 3), and 26 out of 99 prostate cancer-susceptibility loci overlapped with an androgen receptor binding site in prostate cancer cells (Supplemental Table 4). The same bootstrapping approach using random shuffling of the cancer-susceptibility loci around the genome 100,000 times was used to determine the statistical significance of each overlap (Fig. 7). In contradistinction to the overlap with HIF-binding sites in these cancers, the overlap with these tumour-associated transcription factors was significantly more than would be expected by chance in both cases; p = 3 × 10^−4^ for breast cancer-susceptibility loci and ER-binding sites, and p = 5 × 10^−5^ for prostate cancer-susceptibility loci and AR-binding sites.

## Discussion

**Figure 8:**
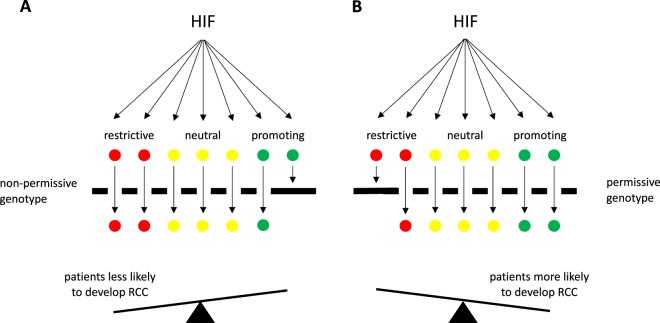
The consequences of HIF activation are modulated by the genotype. The activation of HIF following loss of VHL in ccRCC stimulates the expression of many hundreds of genes that are normally increased in hypoxia. In normoxic RCC cells, these have both beneficial and harmful effects for the cells and the overall consequences of HIF activation are a fine balance of these effects. (**A**) A non-permissive genotype enhances expression of restrictive HIF-target genes rather than tumour-promoting genes and so patients are less likely to develop RCC following HIF activation. (**B**) A permissive genotype enhances tumour-promoting genes over restrictive genes making tumour formation following HIF activation more likely. Variants that affect neutral genes will have no effect on the likelihood of developing RCC and therefore not be under selective pressure. The overall implication is that genetic variants, which increase HIF target genes that specifically promote the development of RCC or which reduce HIF target genes that restrict tumour development will be more abundant in patients with RCC, whereas variants that affect neutral genes will be equally abundant in patients and controls.

Pan-genomic analyses of transcriptional networks have revealed much greater complexity than was foreseen when classical concepts of the role of oncogenes and tumour suppressors in cancer development were first considered. Such analyses of hypoxia signalling pathways led us to consider a model in which potentially oncogenic activation of the extensive HIF transcriptional cascade entrains multiple positive, neutral and negative effects, with the balanced summation of such effects representing the oncogenic output. Under such a model, it might be predicted that multiple events that alter non-neutral components of the HIF transcriptional output would influence cancer development (Fig. 8).

GWAS allows this hypothesis to be tested with respect to human polymorphisms that are present in the population at sufficient prevalence to enable large-scale comparisons with cancer risk^29^. In the current work, we therefore studied a large meta-analysis of GWAS of renal cell kidney cancer^30^ to assess the extent of any co-incidence of risk-associated polymorphisms with genes and regulatory elements in the HIF pathway. We used direct pan-genomic assays of HIF binding by ChIP, assays of chromatin conformation, and assays of gene expression in RCC cell lines to define loci implicated in the HIF transcriptional response. Under each definition, we observed a clear excess in the co-incidence of such loci with RCC-associated polymorphisms defined by GWAS, which was not seen for a control set of polymorphisms associated with educational attainment. These analyses confirmed associations of previously described HIF target loci and RCC-associated polymorphisms, identified several additional loci and demonstrated that the statistical excess overlap with HIF target loci extended to polymorphisms that had not reached levels of statistical significance conventionally applied in GWAS. Taken together, these findings suggest that a large proportion of RCC-associated human polymorphisms act on the HIF pathway.

Based on a summation of the differences between the observed and expected overlap (O-E) for RCC-associated polymorphisms with genotype level P values < 5 × 10^−8^ (conventionally supra-threshold) and the O-E for RCC-associated polymorphisms with genotype level P values < 10^−4^ but > 5 × 10^−8^ (sub-threshold) we estimate that in the region of 10-15 polymorphisms represent real effects on HIF loci on RCC risk. Whilst this is a large proportion of RCC-associated polymorphisms, it is only a small proportion of identified HIF target gene loci. We are not able to distinguish whether this indicates that the large majority of HIF target genes are neutral with respect to RCC development or whether limited human polymorphism or the limited power of the GWAS analysis means that only a proportion of the HIF target gene repertoire was effectively interrogated. Nevertheless, the finding that a large proportion of RCC-associated polymorphisms impinge on the HIF pathway is of interest. RCC is a late-onset disease and multiple behavioural and environment factors (e.g. smoking, obesity, environmental exposure to toxins) have been identified that have no direct impact on hypoxia signalling. The finding that despite all these contributions to aetiology, an unprejudiced survey of human polymorphism, as defined by GWAS meta-analysis, identifies such substantial overlap with a single dysregulated pathway supports the importance of the ‘pathway tuning’ model in the development of RCC.

Inactivation of VHL leads to un-physiological activation of hypoxia signalling pathways and is a common, early and truncal event in RCC^47–49^. Since HIF is upregulated, albeit by different mechanisms, in many cancer types^13^, we also sought to compare these studies of RCC with similar analyses in non-RCC cancers. When identical methodology was used to compare the overlap of HIF-binding sites in cell lines derived from the relevant cancer, and the human polymorphisms associated with that cancer, the overlap was found to be very much more striking for RCC than for other cancers. Indeed, for non-RCC cancer there was no significant excess in overlap. In contrast with these findings, when publicly available data on oestrogen and androgen receptor binding in breast and prostate cancers cell lines was compared with GWAS-defined susceptibility loci for these cancers, highly significant overlap was observed.

We postulate that the contrast between RCC and non-RCC cancer types reflects the contrast between highly un-physiological HIF activation following inactivation of VHL in most RCC, and more physiological activation of HIF by micro-environmental hypoxia in other cancers (i.e. in this setting it is un-physiological dysregulated activation, which creates the selective pressure for modulation). Overall, the observation of multiple overlaps between the *cis*-acting elements of specific transcriptional pathways and GWAS-defined susceptibility loci for specific cancers, suggests that individual transcriptional pathways may often be under selective pressure in a manner that promotes the development of cancer in a specific cell type. Though it is possible that this could be used to focus therapeutic strategies, it is important to recognize that the findings do not, on their own, distinguish whether specific susceptibility loci operate by enhancing positive drive or reducing negative effects or both.

The general implication of the work is that, in cancer development, multiple small effects matter, and importantly that such effects can be defined with respect to specific dysregulated pathways. This is consistent with the emerging picture from cancer genome sequencing studies of remarkably constrained tissue specific patterns of mutations even in pathways that, like the VHL-HIF pathway, have general cellular functions - i.e. somatic mutations that drive cancer must be just right and occur in just the right context for cancer development. Such a model implies that a similar process of ‘pathway accommodation’ should occur by somatic or epigenetic changes during cancer evolution. Whether regions of GWAS-defined susceptibility polymorphism that manifests excess overlap with specific *cis*-acting components of dysregulated signalling pathways can be used to focus the functional interpretation of extra-genic somatic mutation, copy-number variation or dysregulation of epigenetic modifiers that act in *trans*^47^ will be of interest in future work, as the availability of data at whole-genome level increases.

## Methods

### Cell lines

786-O renal cancer cells, and PC3 prostate cancer cells were purchased from ATCC. RCC4, T47D, A549 and HCT-116 were purchased directly from ECACC. Unless used directly from a certified source, the identity of all cell lines was confirmed by STR genotyping and regularly tested for mycoplasma infection. RNA-seq analysis of RCC4 and 786-O cells also confirmed the presence of unique *VHL* gene coding mutations (chr3:10,183,725 C > G and chr3:10,183,841 G > del, respectively). Cell lines were grown in Dulbecco’s modified Eagle’s Medium, supplemented with 100 U/ml penicillin, 100 μg/ml streptomycin and 10% fetal bovine serum (Sigma Aldrich). Primary renal cell cultures were generated from freshly excised ccRCC tissue and tumour-adjacent normal kidney. Briefly, tissue blocks were minced, incubated with 193U/ml Collagenase II and 3.33ug/ml DNase for one hour at 37 °C, with regular pipetting, and then filtered. Cell pellets were resuspended in growth medium (DMEM/F12 1:1, supplemented with glutamax, penicillin-streptomycin, insulin-transferrin-sodium selenite, 4 ng/ml triiodo-L-thyronine, 100 ng/ml epidermal growth factor, 36 ng/ml hydrocortisone and 10% foetal bovine serum). Experiments were performed after the 2^nd^ or 3^rd^ passage.

Hypoxic incubations were performed, as indicated, in an In Vivo2 400 Hypoxia Work Station (Ruskinn Technology).

### ChIP experiments

Chromatin immunoprecipitation (ChIP) experiments were performed as previously described^20^ using the following antibodies: HIF-1α (rabbit polyclonal, PM14)^53^, HIF-2α (rabbit polyclonal, PM9)^54^, HIF-1β (rabbit polyclonal, Novus Biologicals, NB100-110), H3K4me1 (rabbit polyclonal, Millipore, #07-436), H3K4me3 (rabbit monoclonal, Cell Signaling Technology, #9751) or H3K27ac (rabbit poly-clonal, Abcam, #ab4729)^34^. Non-immunized rabbit serum was used as a negative control. qPCR analysis was performed using SYBR green (Life Technologies) and the following oligonucleotides.

**Table:** 

11q13.3	F	CACAGTCACGGACACTGAGG
	R	CCTGGGACACGTACGGC
12p12.1	F	TTTGGAACGGCACCTCTCATT
	R	TGCTGATGGCCTACGTGC
8q24.21	F	TACTTAGCGAGATGTGCCTGC
	R	TTGGAATGCACTTCTGACTTTCTC
3q26.31	F	GGAAACGTGCAGGAAGTCAACA
	R	CATCTCTTGACCTGACCCCGA
12q24.31	F	GATATGCCACATGGAGACGTGA
	R	GCCCTCTTCACAACAGACACAT
15q22.31	F	GCTCCGATCTGGTTTGTCAC
	R	CAGTGCTTAGTCACCGATACCT

### RNA-seq

Total RNA was prepared using the mirVana miRNA Isolation Kit (Ambion; Life Technologies Ltd, Paisley, UK), treated with DNaseI (TURBO DNA‐free, Ambion) and used to generate PolyA + RNA libraries using the ScriptSeq v2 RNA-Seq kit (Epicentre, Madison, WI, USA). In accordance with ENCODE consortium guidelines (https://www.encodeproject.org/documents/cede0cbe-d324-4ce7-ace4f0c3eddf5972/@@download/attachment/ENCODE%20Best%20Practices%20for%20RNA_v2.pdf), all RNA-seq experiments were performed in triplicate.

### Capture-C

Experiments were performed as previously described^34,38^. 3C libraries were generated from 786-O cells using DpnII. Capture enrichment was performed with the SeqCap EZ system (#06953212001, Roche/Nimblegen) using biotinylated oligos (Integrated DNA technologies) as indicated in Supplemental Table 5. A double capture protocol was used^34^.

### High-throughput sequencing

ChIP-seq and RNA-seq libraries were prepared according to standard Illumina protocols. Capture-C libraries were prepared as previously described^34,38^ and sequenced on the HiSeq. 4000 platform (Illumina).

### Statistical analysis

#### ChIP-seq analysis

ChIP-seq datasets were analysed as previously described^25^. Peaks were identified using both the T-PIC (Tree shape Peak Identification for ChIP-Seq)^55^ and MACS (Model-based analysis of ChIP-Seq)^56^. Only peaks identified with both peak callers and present in two or more datasets (i.e. overlapped by at least 1 base pair - BEDTools v2.17.0^57^) were considered. RCC HIF-binding sites were defined by ChIP-seq analysis in two RCC cell lines (RCC4 and 786-O) and were present in a minimum of two out of 5 data sets (RCC4 - HIF-1α, HIF-1β, HIF-2α and 786-O - HIF-1β, HIF-2α).

#### RNA-seq analysis

Analysis of RNA-seq datasets was performed as previously described^21^. HTSeq (0.5.4p3)^58^ with ‘intersection-strict’ mode was used to determine the total read count for each UCSC defined gene. Fold-regulation and statistical significance were determined using DESeq. 2^59^.

#### Capture-C analysis

Capture-C datasets were processed as previously described^38^. CCanalyser2.pl (https://github.com/telenius/captureC/releases) was used to determine interaction frequencies and regions with interaction signals significantly greater than local background were identified as previously described^34^. Proximal and interacting genes were identified using the Ensembl GRCh37 (hg19) reference annotation.

#### Data visualization

Locus-specific data was visualized using Integrative Genomics Viewer (IGV), version 2.3.88^60^. Chromosomal coordinates and gene annotation are from the RefSeq hg19 (GRCh37) build.

#### Overlap of HIF-binding sites and GWAS susceptibility loci

Summary statistics from the RCC GWAS meta-analysis^30^, and from GWAS analyses of educational attainment, breast and lung cancer^32,33,37^, were used to identify independent risk loci. First, the most significant SNP was identified (index SNP) and all SNPs in close physical or genetic distance with reference to the 1000 Genomes^61^ CEU population data (distance < 500 Mb or r^2^ ≥ 0.2) were then excluded. The process was repeated iteratively to identify all independent index SNPs in decreasing order of statistical significance. For prostate and colon cancer no summary statistics of GWAS studies were available and published GWAS loci, reported to the NHGRI-EBI GWAS catalogue (accessed June 2018)^31^, were therefore used. Haplotype blocks at the GWAS loci were defined as the index SNP and all SNPs in high linkage disequilibrium with reference to the 1000 Genomes CEU population (r^2^ ≥ 0.8). The number of loci either directly overlapping or lying within 25 kb of a ChIP-seq peak was recorded. To determine the statistical significance of this overlap, a bootstrapping approach was employed in which the haplotype blocks were randomly moved around the genome and the overlap with ChIP-seq peaks re-assessed. This process was repeated 100,000 times to generate an expected frequency distribution for the overlap and the corresponding p-value for the observed overlap was evaluated. To determine whether HIF-binding sites were enriched compared to non-HIF bound enhancers, the converse analysis, in which the ChIP-seq peaks were randomly moved around regions of the genome defined as active enhancers based on H3K4me1, H3K4me3 and H3K27ac analyses in 786-O RCC cells^36^, was also performed. This process was again repeated 100,000 times to examine the overlap between GWAS-defined susceptibility loci and HIF-binding sites in RCC cells, and the corresponding frequency distribution and p-value evaluated.

#### Gene set enrichment analysis (GSEA)

Weighted GSEA enrichment analysis^62^ used pre-ranked gene lists based on a combined metric incorporating both the fold-difference between the two conditions and the statistical significance of this differential expression, using DESeq. 2, according to the equation^63^.

$${{\rm{\pi }}}_{i}={\varphi }_{i}(-\,lo{g}_{10}p{v}_{i})$$

where **φ**_**i**_ is the log2 fold-change and ***pv***_***i***_ is the p-value for gene *i*.

#### Analysis of TCGA data

To compare expression in ccRCC with normal kidney, gene expression levels were extracted from TCGA KIRC level 3 RNA-seq data for 72 paired renal tumours and surrounding normal renal tissue and compared using a paired Wilcoxon rank sum test. To compare expression levels in ccRCC with other tumour types, gene expression levels were determined from TCGA level 3 RNA-seq data for 6,397 tumours across 25 tumour cohorts. To determine the effect of germline genotype on tumour expression levels, TCGA level 3 RNA-seq expression data for 450 clear cell Renal Cell Carcinoma patients was correlated with Affymetrix Genome-Wide Human SNP Array 6.0 level 2 data on the same tumours as previously described^28^. Briefly, the significance of the association between the SNP genotype and gene expression was determined by fitting RNA-seq expression across patients to a negative binomial Generalized Linear Model (GLM) against the genotype status. The likelihood ratio of this model versus a model that ignores genotype status was then computed and a Chi-Square test used to call significance of the genotype coefficients in stratifying the patients.

## Supplementary information

Supplementary Information

## Data Availability

Accession codes for ChIP-seq data are available from the Gene Expression Omnibus: GSE67237 (HIF-2α and HIF-1β ChIP-seq in 786-O cells); GSE78113 (histone modifications in 786-O cells); GSM1011120 (FAIRE-seq in 786-O cells); GSE120885 (HIF-1α, HIF-2α and HIF-1β ChIP-seq in RCC4 cells); GSE120886 (RNA-seq in RCC4 cells ± VHL); GSE130988 (Capture-C data in 786-O cells), GSE130989 (HIF-1β ChIP-seq in PC3, T47D, A549 and HCT-116); GSE54110 (AR ChIP-seq in PC3 cells treated with R1881); GSM1010823 (ER ChIP-seq in T47D cells treated with BPA).
