# Supplementary material for: Co-incidence of RCC-susceptibility polymorphisms with HIF cis-acting sequences supports a pathway tuning model of cancer
Source: Sci Rep. 2019 Dec 10;9:18768. doi: 10.1038/s41598-019-55098-7 (PMC6904466; doi:10.1038/s41598-019-55098-7)
Supplement: Supplementary file 1 — Supplementary Information [file 41598_2019_55098_MOESM1_ESM.docx]

# Co-incidence of RCC-susceptibility polymorphisms with HIF cis-acting sequences supports a pathway tuning model of cancer

Virginia Schmid^1^, Veronique Lafleur^2^, Olivia Lombardi^2^, Ran Li^2^, Rafik Salama^2^, Leandro Colli^3^, Hani Choudhry^4^, Stephen Chanock^3^, Peter J Ratcliffe^1,5*^, David R Mole^2,6*†^.

## Supplemental Information

**Supplemental Figure 1A**

**Supplemental Figure 1A: 11q13.3** Integrative Genomics Viewer (IGV) tracks at the 11q13.3 locus showing SNP-level p-values from the RCC GWAS meta-analysis (black track), together with ChIP-seq tracks for HIF binding (red tracks), histone modifications (green tracks), and duplicate Capture-C tracks (in blue) of chromatin looping. Capture-C was performed in 786-O cells using “viewpoint” (bait site) oligonucleotides at each active enhancer that coincided with the linked SNPs at the susceptibility locus (r^2^ ≥ 0.8 with the index SNP) as indicated by the red arrows. Regions with Capture-C interaction signals significantly greater than local background are indicated by blue bars and coincide with promoters of the *CCND1*, *ORAOV1* and *MYEOV* genes. The putative gene, *NM_001319657,* also coincides with linked SNPs at this locus. Chromosomal coordinates and gene annotation are from the RefSeq hg19 (GRCh37) build.

**Supplemental Figure 1B**

**Supplemental Figure 1B: 2p21.3** IGV tracks at the 2p21.3 locus as described for supplementary figure 1A. *EPAS1* lies within 25 kb of the LD region at this locus. No statistically significant interactions with more distant gene promoters were identified. However, HIF-1β and HIF-2α signals were observed in a region that was demonstrated in the Capture-C analysis to interact with enhancers close to the *EPAS1* promoter encoding for HIF-2α itself. Note the *PRKCE* promoter lies 300 kb to the left of this window and distant from any capture-C signal.

**Supplemental Figure 1C**

**Supplemental Figure 1C: 12p12.1** IGV tracks at the 12p12.1 locus as described for supplementary figure 1A. *SSPN* and *ITPR2* fall within 25 kb of the LD region, whilst the promoter of *BHLHE41* and an alternate promoter of *ITPR2* (data from ENSEMBL) both coincide with interactions with viewpoint oligonucleotides measured by Capture-C.

**Supplemental Figure 1D**

**Supplemental Figure 1D: 14q24.2** IGV tracks at the 14q24.2 locus as described for supplementary figure 1A. The *DPF3* gene lies within 25 kb of the LD region at this locus. No statistically significant Capture-C interactions with more distant gene promoters were identified.

**Supplemental Figure 1E**

**Supplemental Figure 1E: 12q24.31** IGV tracks at the 12.q24.31 locus as described for supplementary figure 1A. The *SCARB1* gene lies within 25 kb of the LD region at this locus. SNPs at this site are close to a HIF binding site that lies within an enhancer in the first intron of the *SCARB1* gene. Capture-C interactions were observed with the *SCARB1* gene promoter. Although these were not statistically above the local background (likely due to its proximity to the Capture-C “viewpoint”) they likely represent contact with the HIF-bound intronic enhancer. No statistically significant Capture-C interactions with more distant gene promoters were identified.

**Supplemental Figure 1F**

**Supplemental Figure 1F: 8q24.21** IGV tracks at the 8q24.12 locus as described for supplementary figure 1A. Note the HIF-binding site that coincides with this locus shows a statistically significant Capture-C interaction with the *MYC* promoter and with an additional, more distant HIF-binding site that may also link to the *MYC* promoter. The non-coding RNA, *PVT1,* also coincides with this locus.

**Supplemental Figure 1G**

__

**Supplemental Figure 1G: 11q22.3** IGV tracks at 11q22.3 locus as described for supplementary figure 1A. The *EXPH5*, *C11orf65* *KDELC2* genes lie within 25 kb of the LD region at this locus, whilst the promoters of *DDX10*, *EXPH5,* *NPAT* and *ATM* coincide with more distant sites that show Capture-C interaction with the viewpoint oligonucleotides.

**Supplemental Figure 1H**

**Supplemental Figure 1H: 2q22.3** IGV tracks at the 2q22.3 locus as described for supplementary figure 1A. SNPs in LD at this locus overlap the *ZEB2* gene. However, chromatin looping was not assessed since no active enhancers coincided with linked SNPs at this locus.

**Supplemental Figure 1I**

**Supplemental Figure 1I: 1p32.3** IGV tracks at the 1p32.3 locus as described for supplementary figure 1A. *FAF1* and *DMRTA2* lie within 25 kb of the LD region at this locus. No statistically significant Capture-C interactions with more distant gene promoters were identified.

**Supplemental Figure 1J**

**Supplemental Figure 1J: 4q23** IGV tracks at the 4q23 locus as described for supplementary figure 1A. No genes fall within 25 kb of the LD region at this locus and no statistically significant Capture-C interactions with gene promoters were identified at this locus.

**Supplemental Figure 1K**

**Supplemental Figure 1K: 3p22.1** IGV tracks at the 3p22.1 locus as described for supplementary figure 1A. *ENTPD3*, *RPL14* *ZNF619*, *ZNF620* lie within 25 kb of the LD region, whilst the *EIF1B* and *ZNF621* gene promoters coincide with more distant Capture-C interacting regions at this locus.

**Supplemental Figure 1L**

**Supplemental Figure 1L: 15q22.31** IGV tracks at 15q22.31 locus as described for supplementary figure 1A. *DIS3L*, *TIPIN, MAP2K1*, *SNAPC5*, *RPL4* and *ZWILCH* all lie within 25 kb of the LD region at this locus. Statistically significant Capture-C interactions are observed with the promoters of these genes and with the promoters of two more distant genes, *MEGF11* and *SMAD6*.

**Supplemental Figure 1M**

**Supplemental Figure 1M: 3q26.31** IGV tracks at the 31.26.31 locus as described for supplementary figure 1A. The non-coding RNA, *LINC02068,* lies within 25 kb of the LD region, whilst the promoters of *ECT2* and *TNFSF10* coincide with statistically significant Capture-C interacting regions at this locus.

**Supplemental Figure 2**

**Supplemental Figure 2. Overlap between sub-threshold EA-associated GWAS loci and HIF-binding sites and proximity to HIF-induced genes.** Sub-threshold EA-associated loci were defined as independent GWAS loci at which the index SNP failed to reach genome-wide significance (5 x 10^-8^ ≤ p ≤ 1 x 10^-4^). (A) The number overlapping (red arrows) with HIF-binding sites together with the expected overlap (blue bars) was determined as for figure 1. (B) The converse bootstrapping approach was used in which HIF-binding sites were randomly shuffled but constrained to regions defined as active enhancers. (C) The number of sub-threshold loci either overlapping or lying within 25 kb of a HIF-binding site, together with the expected frequency distribution.

## Supplemental Tables

**Supplemental table 1: Breast and prostate cancer-susceptibility loci overlapping HIF-binding sites.**

| **Locus** | **Index SNP** | **p-value** | **3 nearest genes** |
| --- | --- | --- | --- |
| Breast cancer |  |  |  |
| 5q14.2 | rs146817970 | 4.19E-13 | ATG10, ATP6AP1L, RPS23 |
| 17q25.3 | rs8082452 | 1.14E-10 | CBX2, CBX4, CBX8 |
| 8q22.3 | rs514192 | 5.61E-09 | GRHL2, NCALD, ZNF706 |
| Prostate cancer |  |  |  |
| 2p11.2 | rs10187424 | 3.00E-09 | ELMOD3, RETSAT, TGOLN2 |

**Supplemental table 2: Sub-threshold RCC-susceptibility loci (P-value > 5x10^-8^, but < 10^-4^) directly coinciding HIF binding sites.**

| **Locus** | **Index SNP** | **P-value** |
| --- | --- | --- |
| 10q22.1 | rs6480601 | 3.33E-07 |
| 12q23.1 | rs151008469 | 2.23E-05 |
| 15q22.2 | rs11071676 | 6.26E-06 |
| 18q21.2 | rs488155 | 6.11E-05 |
| 19q13.32 | rs6509303 | 5.98E-05 |
| 21q22.3 | rs112658743 | 4.98E-05 |
| 6p22.2 | rs13191296 | 1.94E-05 |
| 6q25.1 | rs2145744 | 3.77E-05 |
| 7p15.3 | rs2285944 | 2.13E-05 |
| 7p21.1 | rs11764350 | 5.03E-05 |

**Supplemental table 3: Breast cancer associated GWAS loci that overlap oestrogen receptor binding sites.**

| **Locus** | **Index SNP** | **Chromosome** | **Position** | **P-value** | **3 nearest genes** |
| --- | --- | --- | --- | --- | --- |
| 2q35 | rs4442975 | chr2 | 217920769 | 1.1E-95 | AC007557.1, TNP1, DIRC3 |
| 3p24.1 | rs60936670 | chr3 | 27332409 | 8.0E-64 | NEK10, SLC4A7, EOMES |
| 16q23.2 | rs7500067 | chr16 | 80648296 | 4.1E-27 | CDYL2, DYNLRB2, CMC2 |
| 8q22.3 | rs514192 | chr8 | 102478959 | 5.6E-09 | GRHL2, NCALD, ZNF706 |
| 16q12.2 | rs2432539 | chr16 | 56420987 | 4.0E-08 | AMFR, GNAO1, NUDT21 |

**Supplemental table 4: Prostate cancer associated GWAS loci that overlap androgen receptor binding sites.**

| **Locus** | **Index SNP** | **Chromosome** | **Position** | **P-value** | **3 nearest genes** |
| --- | --- | --- | --- | --- | --- |
| 19q13.33 | rs11665748 | chr19 | 50851141 | 3.E-54 | NR1H2, NAPSA, KCNC3 |
| 8p21.2 | rs13272392 | chr8 | 23670998 | 4.E-34 | STC1, NKX2-6, NKX3-1 |
| 11q12.3 | rs174549 | chr11 | 61803910 | 2.E-30 | AP003733.1, FTH1, BEST1 |
| 2q37.1 | rs887829 | chr2 | 233759924 | 9.E-25 | NGEF, C2orf82, GIGYF2 |
| 2q31.1 | rs12621278 | chr2 | 172446825 | 9.E-23 | CYBRD1, DYNC1I2, DCAF17 |
| 12q13.13 | rs73110464 | chr12 | 52918828 | 2.E-15 | AC055736.1, KRT5, KRT71 |
| 7p15.3 | rs12155172 | chr7 | 20954872 | 5.E-13 | SP8, ABCB5, ITGB8 |
| 17q24.1 | rs4351 | chr17 | 63492371 | 9.E-13 | AXIN2, CTD-2535L24.2, CEP112 |
| 1q32.1 | rs11568818 | chr11 | 102530930 | 2.E-11 | RP11-817J15.3, MMP27, MMP20 |
| 20q13.2 | rs7582141 | chr2 | 159042977 | 5.E-11 | CCDC148, UPP2, PKP4 |
| 2q24.1 | rs12480328 | chr20 | 50911385 | 5.E-11 | ZFP64, SALL4, ATP9A |
| 8p21.2 | rs11135910 | chr8 | 26034626 | 8.E-11 | PPP2R2A, EBF2, BNIP3L |
| 11p13 | rs4378355 | chr11 | 34761870 | 2.E-10 | EHF, APIP, PDHX |
| 3q21.3 | rs10934853 | chr3 | 128319530 | 3.E-10 | RPN1, C3orf27, GATA2 |
| 10q24.31 | rs3850699 | chr10 | 102654464 | 5.E-10 | FAM178A, PAX2, MRPL43 |
| 7q22.1 | rs6465657 | chr7 | 98187015 | 1.E-09 | NPTX2, BAIAP2L1, BRI3 |
| 12q13.11 | rs80130819 | chr12 | 48025835 | 2.E-09 | RPAP3, ENDOU, RAPGEF3 |
| 18q23 | rs7241993 | chr18 | 79013973 | 2.E-09 | PARD6G, AC139100.2, ADNP2 |
| 3p24.1 | rs481519 | chr3 | 27285723 | 2.E-09 | NEK10, SLC4A7, EOMES |
| 20q11.22 | rs10187424 | chr2 | 85567174 | 3.E-09 | RETSAT, TGOLN2, ELMOD3 |
| 2p11.2 | rs11907546 | chr20 | 34131991 | 3.E-09 | ERGIC3, C20orf173, CEP250 |
| 7p15.2 | rs10486567 | chr7 | 27936944 | 3.E-09 | JAZF1, TAX1BP1, HIBADH |
| 4q24 | rs7679673 | chr4 | 105140377 | 4.E-09 | CXXC4, TACR3, TET2 |
| 5q14.3 | rs35148638 | chr5 | 87315172 | 6.E-09 | TMEM161B, CCNH, RASA1 |
| 6q21 | rs2273669 | chr6 | 108963986 | 8.E-09 | FOXO3, LACE1, ARMC2 |
| 16q22.2 | rs12051443 | chr16 | 71657426 | 3.E-08 | MARVELD3, PHLPP2, TAT |

**Supplemental table 5: Capture oligonucleotide for Capture-C experiments**

| **Genomic Coordinates** | | | **Nearby gene(s)** | **Oligonucleotide Name** | **Sequence** |
| --- | --- | --- | --- | --- | --- |
| chr1 | 50940907 | 50941027 | DMRTA2/FAF1 | chr1:50940907-50941027_AT_1p32.3_1 | /5Biosg/atctagtacctaatttcttacataggctactgggtacatatctgctggcctgggaagccatactttctccaaaagctaaggagagaatggtgtgcagaacaaagaaggactcctgtttgt |
| chr1 | 50941154 | 50941274 | DMRTA2/FAF1 | chr1:50941154-50941274_AT_1p32.3_2 | /5Biosg/ctcagtaacttgtactcatcccatggaaatcctttggaagctacaaaatcaaagacaatctggagcttgttgctggccaggaaacgccgctccaagaactcgccactgggggtccggatc |
| chr1 | 50992473 | 50992593 | DMRTA2/FAF1 | chr1:50992473-50992593_AT_1p32.3_3 | /5Biosg/ccaatgagggttcagatgtctccacatccttgctaacatttgttgttacctgacgactttttttttttttttgagacagagacttgctctgttgcccagctggagtgtagtggcgtgatc |
| chr1 | 50992590 | 50992710 | DMRTA2/FAF1 | chr1:50992590-50992710_AT_1p32.3_4 | /5Biosg/atctcggctcactgcaacgtctgcctctcaggttcaagtgattctcctgcctcagcctcctaagtagctgggactacatgtgcgtgccaccatgcccagcaaagatttttatattttaag |
| chr1 | 50993661 | 50993781 | DMRTA2/FAF1 | chr1:50993661-50993781_AT_1p32.3_5 | /5Biosg/cctcccgagtcgctgggactacaccaccatacttggctaattttttttccctattttttgtagagatggggtttccacatgttgcccaggctggtctcaaactcctgggctcaagtgatc |
| chr1 | 51069440 | 51069560 | DMRTA2/FAF1 | chr1:51069440-51069560_AT_1p32.3_6 | /5Biosg/cttgtagcagggttaaacagcagtttagtcacctcataaaaaagcacactgttaaaaaggaagtgactaataatattctggaaccaacagaatattaaaagacatgtgcaggtcaagatc |
| chr1 | 51086777 | 51086897 | DMRTA2/FAF1 | chr1:51086777-51086897_AT_1p32.3_7 | /5Biosg/atcttggctccatactcctaagctacatgccacagccctggaagtaccaggaagtcccacagctcagggtggggttatggctgtgctggggtttctgatgtgctcctgagtcactgggaa |
| chr1 | 51086931 | 51087051 | DMRTA2/FAF1 | chr1:51086931-51087051_AT_1p32.3_8 | /5Biosg/tgcaaagggggtgctgtgtacacgctcctgcaaggcagctaggaatgggccttcgcaggagccagtgagcaggagggcttgcagatatgccccagtcttacggggaagttagccccgatc |
| chr2 | 46525829 | 46525949 | PRKCE/EPAS1 | chr2:46525829-46525949_AT_2p21_1 | /5Biosg/atctgcccgcctgccctcgcgagcctctcggcactgggtgagaggcaactctggccatttcttgctgccctctcgccctctcccggccgctcccagtccagccgggcccggcgctacccg |
| chr2 | 46526314 | 46526434 | PRKCE/EPAS1 | chr2:46526314-46526434_AT_2p21_2 | /5Biosg/aaccgtgtatcctccggtcgaaagcagcggttcccacctcggggcaccgataaggatttgataaacgggagcgaatcgcgcctgtcctggctcggcgcccgggcccttcactgcgggatc |
| chr2 | 46532980 | 46533100 | PRKCE/EPAS1 | chr2:46532980-46533100_AT_2p21_3 | /5Biosg/atccattatgttgtactcatgattgtggtgctgaaggtaacccccattgccctgtaggctgcccccagaagcagggggtacagccaatagtattcttagaagttaatcaattctgtaacg |
| chr2 | 46534782 | 46534902 | PRKCE/EPAS1 | chr2:46534782-46534902_AT_2p21_4 | /5Biosg/tgtgtggctgggcactgtgtggtcccctgactctctggagtcttccagagttccatggaaattagtgcccccgtgcatttccctgccgactgactggaaggcaccctggctgctgagatc |
| chr2 | 46537855 | 46537975 | PRKCE/EPAS1 | chr2:46537855-46537975_AT_2p21_5 | /5Biosg/atcccaagtcactggagaagattccattccagggaccagtagaggccagcgggtctcatccaagtcactttagctcattctgtccaccggtactttgagggaggctatataactcctgag |
| chr2 | 46547178 | 46547298 | PRKCE/EPAS1 | chr2:46547178-46547298_AT_2p21_6 | /5Biosg/atctattacaacaatctgaataattacagtctctccttcaatttattcaccctggactcctttttctagaattactggccagactcttattacaccaaattccttgactctattcccgag |
| chr2 | 46547554 | 46547674 | PRKCE/EPAS1 | chr2:46547554-46547674_AT_2p21_7 | /5Biosg/gttggcatagcaactcctacgtggaaaagtgcacgccgcttgcagatgacttttgcagtgctcagtgttaatttgataaatggcttttctaatagcagtttgtgggctaatggaaagatc |
| chr2 | 46552302 | 46552422 | PRKCE/EPAS1 | chr2:46552302-46552422_AT_2p21_8 | /5Biosg/atccagtactctccagtgggcagtgctctgtatgttctaggttagagtcttagataatatgggattttaaaaccagtattgtattacattagagtattactgcccttgggaattttttag |
| chr2 | 46552572 | 46552692 | PRKCE/EPAS1 | chr2:46552572-46552692_AT_2p21_9 | /5Biosg/atggtaaaagccctaatagcctcagtgttagaatatcctgtaggcaggcgaaggatgacatgactcactttggtggtgacctcctagtagttcaactgagaatccaggcgggataagatc |
| chr2 | 46558642 | 46558762 | PRKCE/EPAS1 | chr2:46558642-46558762_AT_2p21_10 | /5Biosg/atctttcctctaggtggcatagagttgtggggtttggcagaccaccatgggaaaagaacagatgcatttgtcagaatctctgtggccatcagacgtcactccaatgaagccaaagctccg |
| chr2 | 46559648 | 46559768 | PRKCE/EPAS1 | chr2:46559648-46559768_AT_2p21_11 | /5Biosg/gtgtgtcttgcagggaagtcaaaggcggaggagtagagtagagtggactttttgaagtttgggctaatggacagtgttttttccaaagtgtgggctgttctttagccccacttgctgatc |
| chr3 | 40493835 | 40493955 | ENTPD3/RPL14/ZNF619 | chr3:40493835-40493955_AT_3p22.1_1 | /5Biosg/atcgatagacctggttttgctgactacaggtttgtccctggtggtcttaggctgactgggcttgaacagtcttgctcagcttcagggttcctaagattgcaaatttcaaaaactgataca |
| chr3 | 40494767 | 40494887 | ENTPD3/RPL14/ZNF619 | chr3:40494767-40494887_AT_3p22.1_2 | /5Biosg/tgagagcggtcccaggcggccatcttgattcctggcatcactctcaacgtgccctccctctccgtgattggacgagcagggcgggccttccggaggcacacagccgaccaaccagagatc |
| chr3 | 40499354 | 40499474 | ENTPD3/RPL14/ZNF619 | chr3:40499354-40499474_AT_3p22.1_3 | /5Biosg/cactgtcctttctcctccaattttaggtgttcaggcgcttcgtggaggttggccgggtggcctatgtctcctttggacctcatgccggaaaattggtcgcgattgtagatgttattgatc |
| chr3 | 40518796 | 40518916 | ENTPD3/RPL14/ZNF619 | chr3:40518796-40518916_AT_3p22.1_4 | /5Biosg/acgccctcgaccttcctgctccgaggagctggcctgatgtcctcgggtcgcggtattcatggggaggatgggagccgtgactatagggtctcctgcttcccagggtggagcgttcagatc |
| chr3 | 172309649 | 172309769 | TNFSF10/NCEH1 | chr3:172309649-172309769_AT_3q26.31_1 | /5Biosg/atctgtactgtgggtccaagctggggggttccctggtgacaagcagcaggggtggatgggacctgtgggagatggactggtctcctctccttgggttaactgcagcttgttgggggtgtg |
| chr3 | 172310704 | 172310824 | TNFSF10/NCEH1 | chr3:172310704-172310824_AT_3q26.31_2 | /5Biosg/aggaggactcatccagtgaggagaaacaggtttggagacttgtgtaaaaaagcagtctggccacttttccacaggacagccatgttgtgctggggggtccactccagtcactgatggatc |
| chr4 | 101017255 | 101017375 | H2AFZ/DDIT4L | chr4:101017255-101017375_AT_4q23_1 | /5Biosg/atcaaagccagctttgcctgtgttctttccaccttccagtccccgctgtcctgtgaggataattagcaaacgcagacgtgctcgctcgcacagaacacgctccctctggtggaagtcaga |
| chr11 | 69063141 | 69063261 | MYEOV/CCND1 | chr11:69063141-69063261_AT_11q13.3_1 | /5Biosg/atcccggggccgcctctgtctctcccaggccctgcgtgttgcggtgagaggagcatttgtgtctctgtggtttgctgctggagctggtgaccgggagagaaacaagggagacaagggtgc |
| chr11 | 69234206 | 69234326 | MYEOV/CCND1 | chr11:69234206-69234326_AT_11q13.3_2 | /5Biosg/atcacactggacacaccagattcagacccttccagagccccttccctgctgcagggggcagaaaagaacagataagcgatggtgcatagtggggcagcagctacccccagacttaagaag |
| chr11 | 69234497 | 69234617 | MYEOV/CCND1 | chr11:69234497-69234617_AT_11q13.3_3 | /5Biosg/ggcaacattacatcactttagaccaaagcgacagtgtctgggaagacagagctggttctgcacgtacctccgctccacagtcacggacactgaggtgctcaggggtcggcgctcaggatc |
| chr11 | 69234812 | 69234932 | MYEOV/CCND1 | chr11:69234812-69234932_AT_11q13.3_4 | /5Biosg/atcacatttcacatttgaccaaacgatgttcctctgcagcctccctcccttggtccccaaattggcagcactttctagaagctagatgcagaggatgtcctttgtcaagggaaaaagaag |
| chr11 | 69236338 | 69236458 | MYEOV/CCND1 | chr11:69236338-69236458_AT_11q13.3_5 | /5Biosg/accttgaggctgctgaataccacctgtcccatcactgacacgatgcttccactctgcttattttcttaaataaatgtacttttactggctacttgatagcactgccatatgtggtggatc |
| chr11 | 69251621 | 69251741 | MYEOV/CCND1 | chr11:69251621-69251741_AT_11q13.3_6 | /5Biosg/atcttttggcctagcgtatggggcttgtactccacacgtctcccagggctctgcaggccagcaccggggaccccacccatgttatgctccactttccagggagggaaactgaggctcaca |
| chr11 | 69252082 | 69252202 | MYEOV/CCND1 | chr11:69252082-69252202_AT_11q13.3_7 | /5Biosg/ccttccctccaagccccgcaggtcgcccaactcccagggcggctgcattcactgttcatcccccaggggaagaaaggccgggaccaggggctggccctgggcctgcaccaaccagggatc |
| chr11 | 108337896 | 108338016 | KDELC2/EXPH5 | chr11:108337896-108338016_AT_11q22.3_1 | /5Biosg/atctgtgaaggcgcagtatgagcagtaaggacttgtcaaccagcccgacgcaacttcaccgagggcgaggaaaggagctctgaggaaggggttactcccgggcaggactgtaaaccagct |
| chr11 | 108368505 | 108368625 | KDELC2/EXPH5 | chr11:108368505-108368625_AT_11q22.3_2 | /5Biosg/atccgtcggcttgatgactcaccactggaaagcgctccctgcaatccctcacagttcagacaactaagtccgagatggaaggcaacgtgtgactcacggcacactccttgtaacgcccgg |
| chr11 | 108382270 | 108382390 | KDELC2/EXPH5 | chr11:108382270-108382390_AT_11q22.3_3 | /5Biosg/atcagagttgacggacatctttagattttcacatgaaggtgttcctgactgctctcgtgtagaataattctgtttgtctttttctaaagcgttaggaaatgtttcagtctccacttgagg |
| chr11 | 108382797 | 108382917 | KDELC2/EXPH5 | chr11:108382797-108382917_AT_11q22.3_4 | /5Biosg/ctgaggtcaatggcttttttcttccttctcttccagttgaggcatgtggctcccctgagggacatgacatagccctgttgatgaggaatggaagtggtccttttctaacggaagtagatc |
| chr12 | 26451882 | 26452002 | BHLHE41/SSPN/ITPR2 | chr12:26451882-26452002_AT_12p12.1_1 | /5Biosg/atctcatcttctcttcgatgtttagcctaaggtgattcacttcattcagctttaacatcactaaaatgtgaattcatactattagcgtacttctagtacaagctataaaacccattaccc |
| chr12 | 26452432 | 26452552 | BHLHE41/SSPN/ITPR2 | chr12:26452432-26452552_AT_12p12.1_2 | /5Biosg/agaacctgaaattcaaatctgattattccaggctttactaaaagcatcttgatgacaatgatggtgacattcctcacattctcttgccttatgtaattttaacagctttcatctcggatc |
| chr12 | 26471516 | 26471636 | BHLHE41/SSPN/ITPR2 | chr12:26471516-26471636_AT_12p12.1_3 | /5Biosg/atggggctgaaccactcattccaaaaacttaatatctacaaactcaagcacaaggagcctcttttaattatttggaaactgtgggctgcagggaaagcgagtgctctcagccttgtgatc |
| chr12 | 26492641 | 26492761 | BHLHE41/SSPN/ITPR2 | chr12:26492641-26492761_AT_12p12.1_4 | /5Biosg/atcttgcctgtcacatagctgtcaccatcaccacagtcattttaatagctaccatggttactgttgatatttttactaacaattttatatgataaaggggttcattatagcattgaaatg |
| chr12 | 26493464 | 26493584 | BHLHE41/SSPN/ITPR2 | chr12:26493464-26493584_AT_12p12.1_5 | /5Biosg/tttatacgggcttccagggaggttccggctgagacatttagaaagcaccgtgaaggctgcaggcattcagtgaatgatggtgaatgatgaggtggggtctggaggctgggggagcagatc |
| chr12 | 125322779 | 125322899 | SCARB1/UBC | chr12:125322779-125322899_AT_12q24.31_1 | /5Biosg/atctggccattagggagagggccagggcgcagacctagttctggcctcggctcagtcagcccctcccaccccggtcccctgatatgccacatggagacgtgactgcctccccatcctccc |
| chr14 | 73267875 | 73267995 | DPF3/DCAF4 | chr14:73267875-73267995_AT_14q24.2_1 | /5Biosg/atctgggcaggcagccacagaaacagaagaagctacagaggatgcaggcactgggttttggtgtctggacatggaagaattgcaggccaatatccacaaatgtatccgtgaggctctggt |
| chr14 | 73268300 | 73268420 | DPF3/DCAF4 | chr14:73268300-73268420_AT_14q24.2_2 | /5Biosg/aagtcatcacaaagttctgagtggaagaattcttccagggactgccaaggagtgacactgtagccaggtgggttcagctctcactttgctcaggatatgggtgtccagtggcgacagatc |
| chr15 | 66648115 | 66648235 | TIPIN/MAP2K1/ZWILCH | chr15:66648115-66648235_AT_15q22.31_1 | /5Biosg/atcatgacccactgtaacttcaagcaagctacaagaatctatactagggttcagacctttgaggctgacagcgagctttgagtttgatgacagtacctaaaatatattaagtgtactcag |
| chr15 | 66648600 | 66648720 | TIPIN/MAP2K1/ZWILCH | chr15:66648600-66648720_AT_15q22.31_2 | /5Biosg/ccgtccccctcatcctcgttccccgcctaccctctcttcaacttcattcattcatccaacattcgctgggggatttctacattgacacgccccggacagaagcctggggtaaagatgatc |
| chr15 | 66662187 | 66662307 | TIPIN/MAP2K1/ZWILCH | chr15:66662187-66662307_AT_15q22.31_3 | /5Biosg/atcactgtagctcaaatggtcattctcattttaaatttcctgcttgtaatctttgcaaagcacttggcaggaaacggatgatgcaggggttttccattgagtggaagaaagcatgaggga |
| chr15 | 66662495 | 66662615 | TIPIN/MAP2K1/ZWILCH | chr15:66662495-66662615_AT_15q22.31_4 | /5Biosg/tgagtatatcttctttcactgcgctatccaagggtaaaaagcaaaataccacagtaccacgtctgccaagccagaacgaggtccctaccctgactcccaccaaaaaacaggtgctggatc |
| chr15 | 66679729 | 66679849 | TIPIN/MAP2K1/ZWILCH | chr15:66679729-66679849_AT_15q22.31_5 | /5Biosg/cgacggctctgcagttaacgggaccagctctgcggagtaagtatggggcgggcggtgaacctcggggcccggctggggaggcccgagccggggagcaggagcgcgcgccaggctccgatc |
| chr15 | 66748305 | 66748425 | TIPIN/MAP2K1/ZWILCH | chr15:66748305-66748425_AT_15q22.31_6 | /5Biosg/atctgtcacccctttggggagtgttaagtttctctaaaatctctgggtgacattctgacagtcatgggtaggtacaggtatatgctcctccgtgtctattcgcctacctcctcagtcttt |
| chr15 | 66748860 | 66748980 | TIPIN/MAP2K1/ZWILCH | chr15:66748860-66748980_AT_15q22.31_7 | /5Biosg/tttcagtcagattctcttcactggaggcaggatggacctcactagccccagacacatctagtccttagtgctccctatcccagagaaagtactccacccactctccccagcatccagatc |
| chr15 | 66764854 | 66764974 | TIPIN/MAP2K1/ZWILCH | chr15:66764854-66764974_AT_15q22.31_8 | /5Biosg/atcaccgtggccaggtgacagggagctgtaagtggcccagcctgggtcagggggctgccctgtagccagggacaaggttctcattgtgtcagtctccaccttaagctgcatttgccccaa |
| chr15 | 66765509 | 66765629 | TIPIN/MAP2K1/ZWILCH | chr15:66765509-66765629_AT_15q22.31_9 | /5Biosg/tgttttaatgttattttgtgacctttggcaagagattttatcctccagcctcaacttccatatctgtaaactaggactattctgccaaatattacgaagggtcatgaaaaatgagagatc |
| chr15 | 66774531 | 66774651 | TIPIN/MAP2K1/ZWILCH | chr15:66774531-66774651_AT_15q22.31_10 | /5Biosg/atccacaaacccaggatgctcagatataatccaccccaaactacctcaccttctccttttctcttagcagtaaatagacagcatcatctccctggtttcccaaggtagaaacctgagcat |
| chr15 | 66775143 | 66775263 | TIPIN/MAP2K1/ZWILCH | chr15:66775143-66775263_AT_15q22.31_11 | /5Biosg/ctgggccagtgctgaagtgcggcaaagtggaggagcgttggagtgagtaggcagtctttacagaagtggcggggagtgcaggcccgcgtggagagggggacaggcgcagaggctcagatc |
| chr15 | 66789735 | 66789855 | TIPIN/MAP2K1/ZWILCH | chr15:66789735-66789855_AT_15q22.31_12 | /5Biosg/atccctgagcctcaccctagacttgcgaggtcacgattctgaaagatagggcctaagaagcgcacagcaaactccccaggtgattcagatacctgtttgtttgtaaacctctaaattggc |
| chr15 | 66790184 | 66790304 | TIPIN/MAP2K1/ZWILCH | chr15:66790184-66790304_AT_15q22.31_13 | /5Biosg/aggaaaggcgaggagtgcgcccccttccggcgcccgccgtagcctggccactccgcgggggacgagctgcaggaaggggtacagcctgtgtcaaaacaccttgattcatgagtaatgatc |
| chr15 | 66796512 | 66796632 | TIPIN/MAP2K1/ZWILCH | chr15:66796512-66796632_AT_15q22.31_14 | /5Biosg/atcactgtttaaagccaaaccactcctattccctatgttttcatctcttactaccatagaatattactacgtatttagtccaacgcaactaccatcccagtcaggacagtgtctgttttg |
| chr15 | 66797675 | 66797795 | TIPIN/MAP2K1/ZWILCH | chr15:66797675-66797795_AT_15q22.31_15 | /5Biosg/gatgtgggagcggctgaactgcgcagcagaggacttttattctcgtctccttcagtgagtctagtctcttcttttggctggggtcctgggacagcacttctttccctgggtgtctggatc |
